# Evaluation of the Humoral and Cellular Immune Response Post COVID-19 Infection in Kidney Transplant Recipients

**DOI:** 10.3390/jcm12123900

**Published:** 2023-06-07

**Authors:** Kahina Bensaid, Lydia Lamara Mahammed, Khadidja Habchi, Messaoud Saidani, Ines Allam, Reda Djidjik

**Affiliations:** 1 Immunology Department, Beni-Messous Teaching Hospital, Faculty of Pharmacy, University of Algiers, Algiers 16000, Algeria; 2 Nephrology Department, Beni-Messous Teaching Hospital, Faculty of Medicine, University of Algiers, Algiers 16000, Algeria

**Keywords:** COVID-19, SARS-CoV-2, kidney transplantation, adaptive immunity, interferon-gamma release assay (IGRA)

## Abstract

Kidney transplantation is a major risk factor for severe forms of coronavirus disease 2019 (COVID-19). The dynamics and the persistence of the immune response to severe acute respiratory syndrome coronavirus 2 (SARS-CoV-2) in this immunocompromised population remain largely unknown. This study aimed to evaluate the persistence of humoral and cellular immune response in kidney transplant recipients (KTRs) and to establish whether immunosuppressive therapy influenced long-term immunity in this population. We report here the analysis of anti-SARS-CoV-2 antibodies and T cell-mediated immune responses in 36 KTRs compared to a control group who recovered from mild COVID-19. After a mean time of 5.22 ± 0.96 months post symptom onset for kidney transplant recipients, 97.22% of patients and 100% of the control group displayed anti-S1 immunoglobulin G SARS-CoV-2 antibodies (*p* > 0.05). No significant difference was reported in the median of neutralizing antibodies between the groups (97.50 [55.25–99] in KTRs vs. 84 [60–98] in control group, *p* = 0.35). A significant difference in SARS-CoV-2-specific T cell reactivity was found in the KTRs compared to the healthy controls. The levels of IFNγ release after stimulation by Ag1, Ag2 and Ag3 were higher in the control group compared to the kidney transplant group (*p* = 0.007, *p* = 0.025 and *p* = 0.008, respectively). No statistically significant correlation between humoral and cellular immunity was found in the KTRs. Our findings indicated that humoral immunity persisted similarly for up to 4 to 6 months post symptom onset in both the KTRs and the control group; however, T cell response was significantly higher in the healthy population compared to the immunocompromised patients.

## 1. Introduction

In December 2019, coronavirus disease 2019 (COVID-19) emerged in China, and it has since spread widely across the world and has led to a high death toll. This disease is caused by severe acute respiratory syndrome (SARS-CoV-2) [1].

SARS-CoV-2 is an enveloped, single-stranded positive-sense RNA virus, which is a beta-coronavirus in the family Coronaviridae [1] and is related to two highly pathogenic coronaviruses, SARS-CoV and Middle East respiratory syndrome coronavirus (MERS-CoV) [1,2]. The structure of the virion is composed of four major structural proteins, the nucleocapsid (N), membrane (M), envelope (E) and spike (S), approximately sixteen non-structural proteins (nsp1–16) and five to eight accessory proteins [3,4]. The S protein is inserted in multiple copies into the membrane of the virion, giving it a crown-like appearance [5].

Coronavirus entry into host cells is mediated by the transmembrane spike glycoprotein that comprises two functional subunits: the S1 subunit, which is responsible for binding to the host cell receptor, and the S2 subunit, which is responsible for the fusion of the viral and cellular membrane [5,6]. The entry into host cells is through the receptor angiotensin 2 converting enzyme (ACE2) and the receptor-binding domain of the subunit S1 (S-RBD) [5,7].

As the coronavirus S glycoprotein is surface-exposed, it plays an essential role in viral attachment, fusion, entry and transmission; it is the main target of humoral immune response and antibodies, particularly neutralizing antibodies (Nab), specific to SARS-CoV-2 [7,8]. The cellular immune response is directed against almost all SARS-CoV-2-proteins [9].

In healthy recovered patients, studies of the humoral immunity against SARS-CoV-2 have reported a persistence of IgG anti-S levels as well as neutralizing antibodies for 6 to 13 months following infection [10,11,12,13]. Additionally, several studies indicate that COVID-19 infection generates long-lasting B cell memory for up to 8 months post-infection [11,14,15]. Moreover, investigations of cellular immunity indicate the persistence of functional SARS-CoV-2-specific T cells (CD4+ and CD8+) for up to 12 months following infection [16,17].

Despite the studies on SARS-CoV-2-induced immunity, little is known about the magnitude and longevity of protective immunity against the virus in immunocompromised populations and whether this is similar in immunocompetent patients. Among these immunocompromised patients, kidney transplant recipients have been shown to be particularly concerning in view of the severity of the COVID-19 infection [18], because of long-term exposure to immunosuppressive therapy that includes drugs targeting T cells, such as corticosteroids and calcineurins inhibitors, which may impair immune functions and predispose these patients to severe forms of COVID-19 [19].

This has raised several questions about the protective role of humoral and cellular immunity in this population. The kinetics of the antibodies showed that a substantial proportion of kidney transplant recipients had a stable titer of IgG antibodies 6 months after infection [20], as well as the T cell immune response [21]. However, little is known about the magnitude of both humoral and cellular immune responses compared to healthy COVID-19-infected subjects.

Therefore, the aim of our study is to better characterize the persistence of humoral and cellular immune response in kidney transplant patients compared to healthy COVID-19-infected subjects and to understand whether immunosuppressive therapy influences long-term immunity in this population.

## 2. Materials and Methods

### 2.1. Study Population

The prospective study was conducted at the department of medical immunology at ISSAD HASSANI hospital, Algiers, Algeria, in the period from June 2022 to September 2022.

The case control study included two groups:

- A study group composed of 36 adult kidney transplant recipients (the mean age was 35.90 ± 7.68 years and 61.11% were male);
- A control group composed of 35 health-care subjects (the mean age was 34 ± 8.72 years and 45.71% were male), none of whom had comorbidities or had received immunosuppressive treatment before.

All the participants of the study were aged between 18 and 65 years and diagnosed with mild COVID-19 infection based on their clinical symptoms and positive antigen test results obtained using nasopharyngeal swabs. Patients exhibited mild symptoms (e.g., fever, cough, sore throat, headache, anosmia and ageusia), did not have shortness of breath, dyspnea or abnormal imaging and were ambulatory treated. None of these subjects had received a COVID-19 vaccine previously.

Demographical, clinical and biological data were collected from patient records (Table 1).

The study was approved by the institutional ethics committee of Beni-Messous university hospital center, Algiers, Algeria. All participants gave written informed consent for possible subsequent uses of their samples for research.

### 2.2. Methods

Blood samples were collected for the evaluation of humoral immunity (SARS-CoV-2-specific antibodies) and cellular immunity (T cell response).

#### 2.2.1. SARS-CoV-2 Serological Assessment

##### Measurement of SARS-CoV-2-Specific Antibodies

AntiSARS-CoV-2 antibodies were evaluated using an in vitro chimiluminescence immunoassay for the quantitative determination of S-RBD IgG antibodies to SARS-CoV-2 in human serum using the SNIBE analyzer MAGLUMI 800. Values were expressed as arbitrary units per mL AU/mL. According to the manufacturer’s recommendations, a result greater than or equal to 1 AU/mL was considered as positive. Samples with values greater than 100 AU/mL were diluted to 1/10.

##### Detection of Neutralizing Antibodies against SARS-CoV-2

Neutralizing antibodies against SARS-CoV-2 (Nab) were evaluated using the enzyme immunoassay ELISA (EUROIMMUN, Lubeck, Germany). This assay allows a semi-quantitative determination of antibodies that inhibit the binding of S-RBD to ACE2 receptors of human cells. Neutralizing antibodies can inhibit the receptor-mediated entry of the virus into the host cells. Values were given in percentage of inhibition (%IH). A result above 35% was interpreted as positive.

#### 2.2.2. Evaluation of Cellular Immune Response

To provide a detailed characterization of the cellular SARS-CoV-2 immune response, a SARS-CoV-2-specific interferon-γ release assay (IGRA) was applied (QuantiFERON^®^ SARS-CoV-2 assay (Qiagen, Hilden, Germany)). The principle of this test consists of a stimulation of CD4+ and CD8+ T cells by SARS-CoV-2 antigens (Ag1, Ag2 and Ag3) contained in three blood collection tubes.

- Epitopes of Ag1 are derived from the S1 subunit (receptor-binding domain) of the spike protein and stimulate CD4+ T cells.
- Epitopes of Ag2 are obtained from the S1 and S2 subunits that stimulate CD4+ and CD8+ T cells.
- Ag3 epitopes are derived from S1, S2 subunits, from N and M domain and from whole genome. These epitopes activate both CD4+ and CD8+ T cells.

The test was performed in the presence of a positive (mitogen) and a negative (nil) control. The mitogen tube serves as a control for correct blood handling and incubation.

Following 16 h incubation period at 37 C, the stimulation of CD4+ and CD8+ T cells induced the release of IFN-γ that were quantified using an enzyme-linked immunosorbent assay (ELISA) according to QuantiFERON SARS-CoV-2 instructions (Germantown, MD, USA).

For analysis, data from the negative control tube was subtracted from the signal obtained after stimulation with peptides. IFNγ response was expressed in IU (International Units)/mL and a result above 0.15 IU/mL was considered as positive.

### 2.3. Statistical Analysis

Categorical variables were expressed as frequencies and percentages and were compared using the Fisher exact or the chi-squared test. For the test of normality, a Shapiro–Wilk test was conducted to estimate the distribution of the continuous variables, where *p* < 0.05 indicated that the level of S-RBD IgG, Nab and the IFNγ release were not normally distributed. Continuous variables were presented as median and interquartile range [first interquartile–third interquartile]. Differences between two independent groups were analyzed using the nonparametric Mann–Whitney U Test and the comparison of quantitative variables in multiples independent groups was calculated using the Kruskal–Wallis test. Correlations between quantitative variables were determined using the Spearman rank correlation analysis. To perform all the analyses, the software SPSS v.25 (IBM Corp, Armonk, NY, USA) has been used and *p* < 0.05 was considered statistically significant for all analyses.

## 3. Results

**Figure 1:**
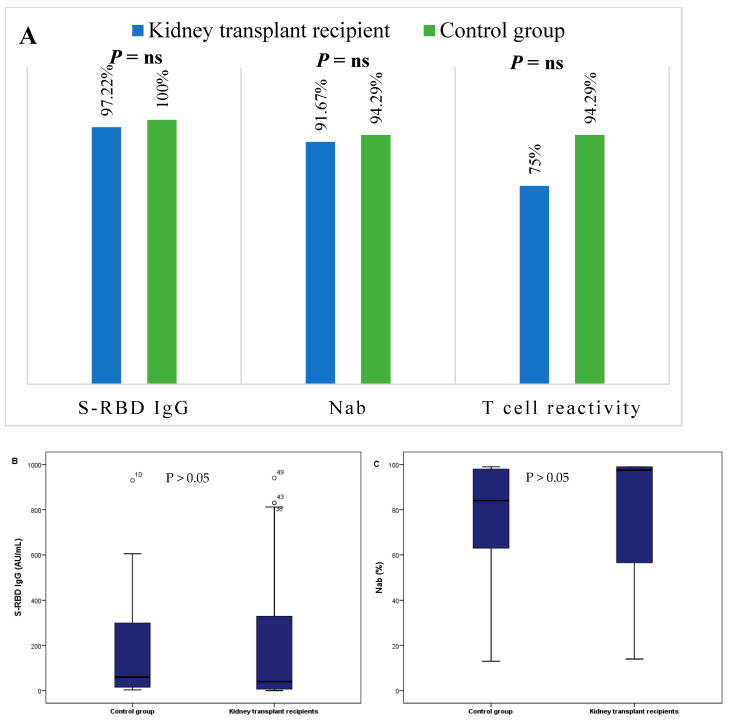

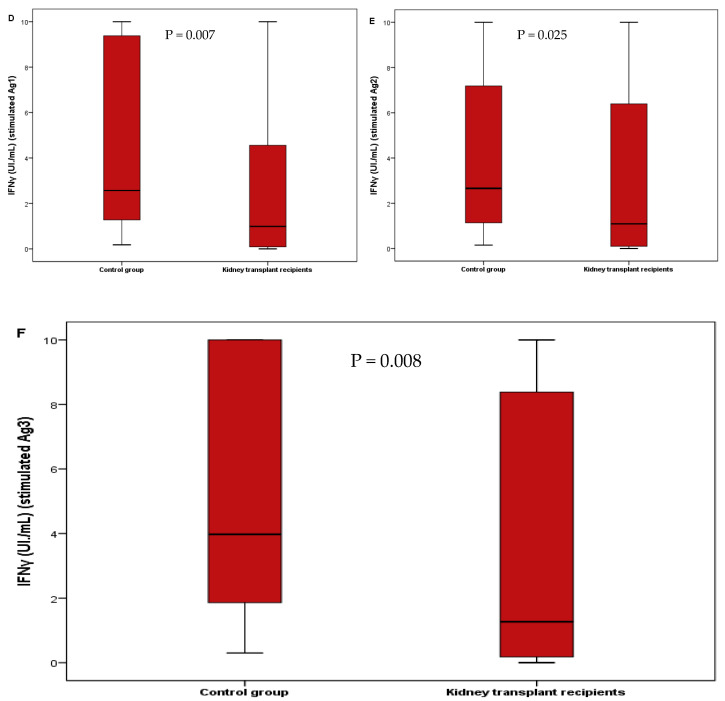
Comparison of humoral and cellular immune responses to SARS-CoV-2 between kidney transplant patients and control group. (**A**) Rates of positivity of S-RBD IgG, neutralizing antibodies and IFNγ. (**B**) Levels of S-RBD. (**C**) Neutralizing antibody levels. (**D**) IFNγ levels after stimulation by Ag1. (**E**) IFNγ levels after stimulation by Ag2. (**F**) IFNγ levels after stimulation by Ag3. IFNγ, Interferon gamma; S-RBD, Spike protein receptor-binding-domain; Nab, Neutralizing ab.

In this study, the analysis of the humoral immune response showed a frequency of S-RBD IgG and neutralizing antibodies of 97.22% and 91.67%, respectively, in the kidney transplant group compared to 100% and 94.29% in the control group. The statistical analysis did not show any significant difference (Figure 1A). A positive IGRA test (defined by a reactivity to at least one antigen) was observed in 75% and 94.29% of kidney transplant patients and the healthy group, respectively, but was not statistically significant (Figure 1A).

We also observed no significant difference in levels of S-RBD (39.89 [6.70–363.25] in kidney transplant recipients vs. 59.87 [13.93–333] in control group (*p* = 0.696)) and Nab (97.5 [55.25–99] in kidney transplant recipients vs. 84 [60–98] in control group (*p* = 0.355)) (Figure 1B,C).

However, we noticed a significant difference in cellular immune response between the two groups; levels of the IFNγ release after stimulation by Ag1, Ag2 and Ag3 were higher in the control group compared to the kidney transplant group (2.57 [1.26–10] vs. 0.99 [0.09–4.65] (*p* = 0.007)), (2.66 [1.12–8.39] vs. 1.09 [0.09–7.45] (*p* = 0.025)) and (3.97 [1.77–10] vs. 1.27 [0.16–8.51] (*p* = 0.008)), respectively (Figure 1D–F).

To define factors influencing the immune response after COVID-19 infection, differences in the humoral and cellular response between the different groups were analyzed. We analyzed the immune response according to different factors: comorbidities, history of transplant rejection, lymphopenia and immunosuppressive therapy (Table 2).

The results in Table 2 show that the median levels of IFNγ released after stimulation by Ag1, Ag2 and Ag3 were higher in patients who did not have any episodes of rejection compared to those who had a rejection episode, but the differences were not statistically significant (2.85 [0.48–10] vs. 1.29 [0.79–4.45]; *p* = 0.557) for Ag1, (2.38 [0.89–10] vs. 1.55 [0.89–8.88]; *p* = 0.713) for Ag2 and (3.24 [0.52–10] vs. 1.56 [1.09–8.59]; *p* = 0.607) for Ag3. The same tendency was observed for the median levels of anti-SARS-CoV-2 antibodies (39.98 [13.14–262] vs. 7.25 [3.71–660.5]; *p* = 0.391) for S-RBD and (98 [72–99] vs. 75 [34–99]; *p* = 0.576) for Nab. Moreover, the statistical analysis between patients receiving immunosuppressive tritherapy (CNI+MMF+CS] compared to patients who received immunosuppressive bitherapy (CNI+MMF or CNI+CS) showed no significant difference. The comparison according to comorbidities and lymphopenia showed no significant difference.

**Figure 2:**
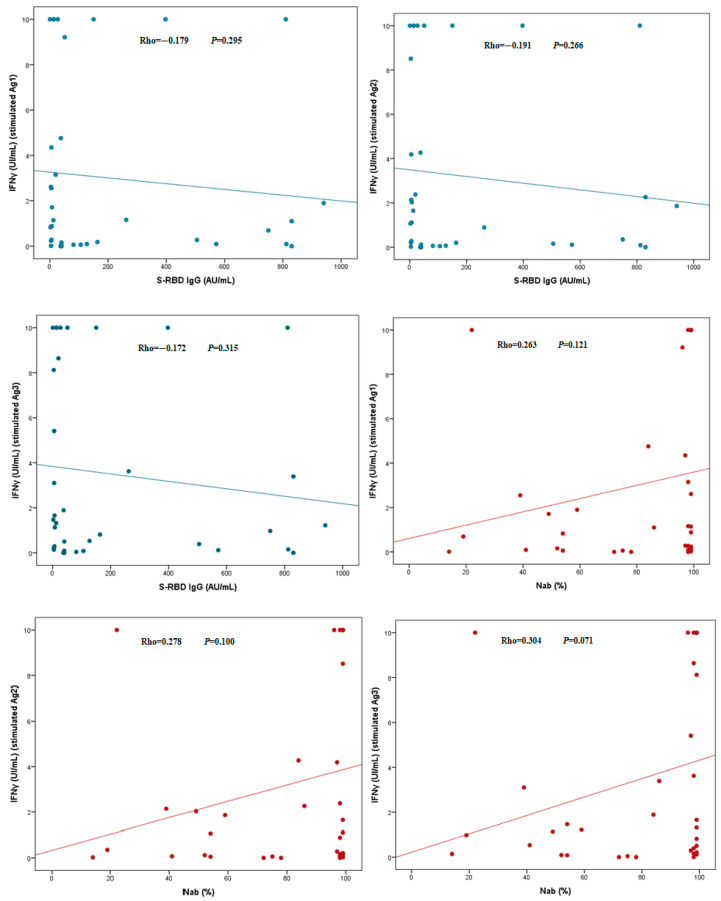
Correlation between humoral and cellular immune response in kidney transplant patients. IFNγ, Interferon gamma; S-RBD IgG, IgG against the receptor-binding domains of the subunit S.

Additional evaluation of the humoral and cellular immune response of the kidney transplant patients showed that there was no correlation between the levels of anti-SARS-CoV-2 antibodies (S-RBD IgG and Nab) and the levels of IFNγ release (Figure 2).

## 4. Discussion

The severe acute respiratory syndrome coronavirus 2 caused a rapidly growing outbreak of coronavirus disease and a global pandemic. Consequently, a significant proportion of kidney transplant recipients were infected, and whether these patients are capable of mounting an effective adaptive immune response despite chronic immunosuppression is unknown and has important implications for therapy. The aim of our study was to analyze the humoral and cellular immune responses to SARS-CoV-2 in kidney transplant recipients and healthy recovered subjects over the late convalescent clinical course after COVID-19 infection.

We evaluated SARS-CoV-2 antibodies levels in both KTRs and the control group. Our results showed that humoral immune response to SARS-CoV-2 in kidney transplant recipients was comparable to the immunocompetent cohort, and nearly all the kidney transplant patients were able to produce SARS-CoV-2 antibodies (97.22% had IgG anti-S-RBD and 91.67% had Nab). Indeed, Fava et al. reported no differences regarding both seroconversion rates and IgG titers against antigen S between solid organ transplant and immunocompetent individuals [22,23]. More interestingly, a higher IgG level was found in kidney transplant recipients compared to immunocompetent subjects and the presence of moderate to severe symptoms was associated with higher levels of IgG antibodies in the population of symptomatic KTRs as compared to asymptomatic KTRs [24]. Several studies in the literature have reported that transplant patients produce antibodies against SARS-CoV2 within 2 months after infection [18,25,26]. Taken together, the present findings suggest that transplant patients have, in the same way as immunocompetent patients, the ability to build a protective and effective humoral immune response.

According to our findings, antibodies against SARS-CoV-2 persisted at least 6 months following infection in the immunocompromised patients in a similar way to that seen in the immunocompetent group. These results are in accordance with the studies of Bajpai et al. and Benotmane et al., who reported that a subsequent proportion of KTRs developed a serological response that was sustained for up 6 months after COVID-19 infection [20,26]. The same trend has also been observed for other transplant groups, where no significant difference in the proportions of positivity of IgG titers against antigen S have been reported when compared to immunocompetent individuals 6 and 9 months after infection [23,27]. Correspondingly, some authors have shown that solid organ transplant patients are capable of maintaining specific SARS-CoV-2 memory B cells in a similar way to immunocompetent individuals, which can provide a long-term humoral protective immunity even in the absence of detectable antibodies in the blood. However, the persistence and magnitude of this response is mainly influenced by the degree of COVID-19 clinical severity [23]. Nevertheless, N Chavarot et al. reported that the humoral response kinetics decline and serum antibody levels decrease more rapidly compared with immunocompetent subjects [25].

We then evaluated the cellular immune response against SARS-CoV-2 peptides using an IFNγ QuantiFERON assay. Our results showed that both immunocompromised patients and the control group responded to peptide stimulation 6 months after infection (75% of kidney transplant recipients vs 94.29% of the healthy group). M Fernández-Ruiz et al. reported a persistent T cell immunity (CD4+ and CD8+ T cells) reactive to the S1 subunit for at least 6 months in 21 kidney transplant recipients recovered from moderate to severe COVID-19 [28]. In the study of D Bertrand et al., the T cell response persisted for up to 3 months post-symptom onset in the majority of kidney transplant recipients and could still be detected 10 months after infection for some patients [21]. In fact, five KTRs were evaluated in the late recovery stage from SARS-CoV-2 infection: T cell numbers remained stable in 3 patients and decreased, but were still detectable, in the 2 others, indicating that T cell response could still be present and could probably provide a long-term protective immunity (none of the patients of the cohort experienced reinfection) [21].

However, when comparing the levels of IFNγ between the two cohorts, we noticed that they were significantly higher in the control group. Our findings are in agreement with those of D Kamińska et al., who reported a significantly higher IFNγ response in hemodialysis patients compared to kidney transplant ones [29]. The cellular immune response post vaccination is also quite different. Affeldt et al. reported that the cellular immune response after two vaccinations vanished rapidly within 2–3 months in kidney transplant patients compared to a healthy control group [30]. In addition, J Stumpf evaluated T cell response after the first and the second vaccination in 368 kidney transplant recipients compared to 144 controls and reported that the IFNγ release values were overall lower in KTRs compared to healthy and dialysis patients [31]. However, our results are different from those of A Favà et al., who showed that solid organ transplant patients (38 kidney transplant recipients among 53 solid organ transplant patients) were capable of maintaining a long-lasting functional T cell immune response specific to SARS-CoV-2, similarly to immunocompetent individuals [23]. In addition, as for memory B cells, the most relevant feature determining the persistence and magnitude of protective immunity was the degree of COVID-19 clinical severity [23].

The KTRs infected with COVID-19 may have an increased risk of severe infection than the general population [32]. In order to study the impact of immunosuppression as well as other factors on the immune response, we analyzed the magnitude of both cellular and humoral response in kidney transplant patients according to comorbidities, history of rejection, lymphopenia and immunosuppressive therapy.

The use of immunosuppressive medications is considered as a factor associated with severe forms of COVID-19 [33]. Some European studies have shown that mortality rates increase significantly when SARS-CoV-2 infection occurs in the first period following kidney transplantation, suggesting that the intensity of immunosuppression may impact outcomes [33]. A Spanish study that included 90 kidney transplant patients who received two doses of the mRNA-1273 vaccine (Moderna), indicated that a short time after transplantation, which equates to a higher immunosuppression burden, is the most important factor responsible for reduced mounting of an anti-SARS-CoV-2 immune response within the first post-transplant months [34]. In our study, the mean time after transplantation was 6.14 ± 3.32 years, so the immunosuppression-related drawback in a specific immune response may not have been pronounced. Nevertheless, we observed that median of IFNγ release was higher in patients receiving immunosuppressive bitherapy (CNI+MMF or CNI+CS) compared to patients who received immunosuppressive tritherapy (CNI+MMF+CS) even if the statistical difference was not significant.

Moreover, we did not find any significant difference in either humoral or cellular response according to comorbidities and lymphopenia. Lymphopenia is a common feature of SARS-CoV-2 infection and leads to reduced effector functions [33]. This is not in line with David Cucchiari et al., who reported that diabetes and lymphopenia were factors associated with absence of cellular response to the S protein in kidney transplant patients after mRNA-1273 (Moderna) vaccination [35]. These differences could be linked to the small sample size of each sub-group, and an increase in the number of recruits may show a statistically significant result.

The study of the correlation between SARS-CoV-2 T cell reactivity and antibody titers in kidney transplant patients with confirmed COVID-19 did not show any significance. This is in line with results of S Candon and D Kaminska, who reported the same observation [29,36]. These findings could be explained by an impaired cellular immune response following chronic immunosuppression in immunocompromised patients [36]. In the study of A Fava et al., the author associated this disparity in the response to the severity of the infection and that transplant patients showed a rapid decline in the memory T cell immune response, illustrating the impact of chronic immunosuppression on the antiviral immune response [23]. The adaptive immune response induces the generation of effectors that lead to the elimination of viral particles and infected cells. Cytotoxic TCD8+ cells are capable of inducing cell death through the excretion of perforin and serine proteases. TCD4+ helper provides help to lymphocytes B inducing antibody production by plasma cells. Therefore, an effective adaptive immune response has an important role in viral clearance [33].

There are some limitations in this study, such as the small sample size evaluated, which does not allow us to draw solid conclusions about the humoral and cellular immune responses. The variability of the timing from symptom onset to the immune response assessment did not allow us to determine the persistence of both antibodies and T cell responses. Finally, the immunosuppressive dosing regimen was not taken into account and this could influence the response, especially the T cell immune response.

## 5. Conclusions

In conclusion, we reported here specific humoral and cellular immunities in KTRs 4 to 6 months from symptom onset. SARS-CoV2-specific antibodies remained positive in a large majority of kidney transplant patients, similarly to the control group. However, T cell reactivity was significantly higher in the healthy population compared to the immunocompromised patients. Our findings have implications for the understanding of immune response to COVID-19 infection in immunocompromised patients.

## Figures and Tables

**Table 1:** Demographical, clinical and biological characteristics of KTR and control group.

Variable	Kidney Transplant Recipients (*n* = 36)	Control Group (*n* = 35)
**Age (years) mean ± SD [extremes]**	35.9 ± 7.68 [21–57]	34 ± 8.72 [20–55]
**Gender**		
- Male, n (%) - Female, n (%)	22 (61.11) 14 (38.89)	16 (45.71) 19 (54.29)
**Comorbidities**		
No, n (%)	13 (36.11)	35 (100%)
Yes, n (%)	23 (63.89)	
- Hypertension, n (%) - Diabetes, n (%)	22 (95.65) 1 (4.35)	
**Cause of end-stage renal disease**		
Hypertension, n (%)	1 (2.78)	
Glomerulonephritis, n (%)	1 (2.78)	
Renal malformation, n (%)	4 (11.11)	
Unknown, n (%)	30 (83.3)	
**Time from transplantation (years) mean ± SD [extremes]**	6.14 ± 3.32 [0.6–14]	
**Immunosuppressive therapy**		
CNI only, n (%)	2 (5.56)	
CNI+MMF, n (%)	12 (33.33)	
CNI+CS, n (%)	6 (16.67)	
CNI+MMF+CS, n (%)	16 (44.44)	
**History of rejection, n (%)**	9 (25)	
**Biological parameters**		
- Lymphocytes (/mm^3^), mean± SD - Lymphopenia (<1000/mm^3^), n (%) - eGFR (mL/min/1.73 m^2^), mean± SD - Urea (g/L), mean± SD - Serum creatinine (mg/L), mean± SD	5975 ± 1564 7 (19.44) 814.3 ± 63.67 0.45 ± 0.17 15.96 ± 5.54	




**Time from COVID-19 disease (months) mean ± SD**	5.22 ± 0.96	

SD, Standard deviation; CNI, Calcineurin-inhibitors; MMF, Mycophenolate mofetil; CS, Corticosteroids; eGFR, estimated glomerular filtration rate.

**Table 2:** Study of the association of comorbidities, history of rejection, lymphopenia and immunosuppressive therapy with humoral and cellular immune response.

		Comorbidities			History of Rejection			Lymphopenia			Immunosuppressive Therapy			
		Yes *n* = 23	No *n* = 13	*p*	Yes *n* = 9	No *n* = 27	*p*	Yes *n* = 7	No *n* = 29	*p*	CNI+MMF *n* = 12	CNI+CS *n* = 6	CNI+MMF+CS *n* = 16	*p*
**Humoral immune response**	RBD median ± IQR	27.11 [6.04–150]	127 [29.75–790]	0.070	7.25 [3.71–660.5]	39.98 [13.14–262]	0.391	127 [5.96–361.16]	39.80 [6.88–383.5]	0.920	94.99 [29.51–527.5]	35.21 [9.1–667.5]	25.45 [4.40–154]	0.666
	Nab median ± IQR	98 [78–99]	75 [46.5–98]	0.077	75 [34–99]	98 [72–99]	0.576	99 [44.25–99]	97 [56.5–99]	0.262	98 [82.75–99]	97 [62.5–98.5]	79.5 [49.75–99]	0.543
**Cellular immune response**	Ag1 median ± IQR	1.71 [0.18–10]	0.27 [0.07–1.5]	0.098	1.29 [0.79–4.45]	2.85 [0.48–10]	0.557	10 [1.72–10]	1.71 [0.48–6.98]	0.236	10 [4.35–10]	5.88 [1.46–9.80]	0.48 [0.10–1.85]	0.389
	Ag2 median ± IQR	2.03 [0.2–10]	1.86 [0.35–2.38]	0.105	1.55 [0.89–8.88]	2.38 [0.89–10]	0.713	10 [4.79–10]	2.08 [0.48–8.56]	0.213	10 [4.19–10]	2.26 [1.15–10]	1.11 [0.28–4.27]	0.344
	Ag3 median ± IQR	3.62 [1.13–10]	1.22 [0.51–6.01]	0.145	1.56 [1.09–8.59]	3.24 [0.52–10]	0.607	8.12 [0.53–10]	1.89 [0.89–9.32]	0.127	10 [4.06–10]	3.39 [1.74–10]	0.89 [0.16–1.61]	0.192

## Data Availability

The datasets generated and analyzed during the current study are not publicly available due to health privacy concerns, but are available from the corresponding author on reasonable request.
